# Estimation of the burden of people living with Human Immunodeficiency Virus/Acquired Immunodeficiency Syndrome (HIV/AIDS) in Kerala state, India

**DOI:** 10.3126/nje.v8i3.23752

**Published:** 2018-09-30

**Authors:** Brijesh Sathian, Jayadevan Sreedharan, Mohammad Asim, Ritesh G Menezes, Edwin van Teijlingen, Bhaskaran Unnikrishnan

**Affiliations:** 1 Academic Research Associate, Clinical Research, Trauma and Vascular Surgery, Surgery Department, Hamad General Hospital, Doha, Qatar.; 2 Professor of Epidemiology and Biostatistics, College of Medicine, Gulf Medical University, Ajman, UAE; 3 Professor, Department of Pathology, College of Medicine, King Fahd Hospital of the University, Imam Abdulrahman Bin Faisal University, Dammam, Saudi Arabia (KSA); 4 Professor, Centre for Midwifery, Maternal and Perinatal Health, Bournemouth University, Bournemouth, UK.; 5 Associate Dean and Professor, Department of Community Medicine, Kasturba Medical College (Affiliated with Manipal Academy of Higher Education), Mangalore, India

**Keywords:** Incubation time, Human Immunodeficiency Virus, AIDS, statistical modelling, Kerala

## Abstract

**Background:**

Worldwide, 36.7 million people were infected with Acquired Immunodeficiency Syndrome (AIDS) by the end of 2015. Over the period 2007 to 2015, there was a declining trend in the prevalence of adult Human Immunodeficiency Virus (HIV) in the state of Kerala, India. The current study aims to find a suitable statistical modelling technique for the distribution of HIV incubation time and predict the cumulative number of AIDS cases.

**Materials and Methods:**

The requisite data were obtained from the Kerala State AIDS Control Society (KSACS) for the years 2007 to 2015. To assess the distribution of HIV incubation time, the data of 22 HIV-infected Keralite patients were retrieved from the medical records of a teaching hospital. Data included age, gender, and incubation time. The back-calculation method was utilized to predict the cumulative HIV/AIDS cases.

**Results:**

The estimated total cumulative AIDS cases in Kerala for the years 2005, 2006, 2007, 2008, 2009, 2010, 2011, 2012, 2013, 2014 and 2015 were found to be 35,777, 48,944, 62,039, 45,669, 45,668, and 43,605, 42,377, 39,362, 37,617, 39,583, 25,414 respectively using back-calculation method with Weibull (2) incubation time distribution. The mean incubation time of the total HIV cases (male and female) was 4.4 years which indicates a rapid progression of the disease in the state of Kerala.

**Conclusion:**

The back-calculation method is a powerful tool to estimate the cumulative frequency of AIDS cases; which predicted a declining HIV trend among Keralites. Moreover, the Weibull distribution is the best fitted distribution for HIV incubation time in our population.

## Introduction

The global epidemic of Human Immunodeficiency Virus (HIV) infection has varied markedly between the developed and developing countries, depending on the socio-cultural characteristics and behavioural patterns. Especially in India, the scenario of HIV infection has changed considerably since the first HIV/AIDS case was identified in Tamil Nadu in 1986. The prevalence of HIV in India has declined from 0.41% in 2000 to 0.31% in 2009 [1]. Based on the latest estimates from National AIDS Control Organization (NACO) there is a slight decrease in the total number of people living with HIV (PLHIV) in India from 22.26 lakhs (= 2.23 million /range 18.00-27.85 lakhs) in 2007 to 21.17 lakhs (17.11– 26.49 lakhs) in 2015 [2]. In general, most of the Indian states, including Kerala, showed a declining trend in HIV prevalence [2]. There are numerous epidemiological parameters of HIV/AIDS and each of them has specific definitions, but it is worth remembering that much of the data are estimates only. In 2001, the Joint United Nations Programme on HIV/AIDS (UNAIDS) and World Health Organization (WHO) introduced the Estimation and Projection Package (EPP) for the estimation of HIV/AIDS which utilizes the surveillance data from the respective sentinel site for HIV [3]. However, the generalisability of such estimates remains doubtful because of the poor vital registration systems in HIV sentinel sites [2].

Therefore, accurate estimates on HIV prevalence are crucial for an efficient and effective response to the epidemic. The application of statistical modelling approaches confers invaluable contribution for developing better understanding of the burden and trends of the HIV epidemic. The literature suggested four different modelling approaches used to develop mathematical models for AIDS: (1) stochastic; (2) deterministic; (3), state space; and (4) statistical model [3-28]. Although, several statistical and mathematical approaches are available to forecast the future course of the disease based on AIDS epidemiological and survey data; most require certain assumptions based on extrinsic and intrinsic dynamics of the disease spread [3-11]. Therefore, ideal model building necessitates specialized epidemiological knowledge and expertise. Unfortunately, most of the non-experts in this field directly adopt the prevalence of HIV, thereby neglecting the significant flaws and key assumptions of the disease spread. Therefore, the present study aims to find a suitable statistical modelling technique for the distribution of HIV incubation time and predict the cumulative number of AIDS cases.

## Methodology

### Study design

It is a cross-sectional study to find out the distribution of HIV incubation time using data obtained from the medical records of 22 Keralite HIV patients treated at Mangalore and the annual reported number of HIV cases was collected from the Kerala State AIDS Control Society (KSACS) for forecasting the number of AIDS cases.

### Data collection

The data were obtained from the hospital records at an affiliate teaching hospital of Kasturba Medical College, Mangalore, India. Mangalore city is situated in the neighbouring state of Karnataka and is less than 20 kms away from the northern border of Kerala. Although, geographically this city is not in the state of Kerala, many patients from the northern districts of Kerala visit Mangalore for medical treatment. The requisite data of the annual reported number of HIV cases was collected from the KSACS for the years 2005 to 2015.

### Sample size calculation

The sample size was not determined priori, as we intended to include all reported number of AIDS cases per year during the study period.

### Outcome variable

Cumulative number of AIDS cases was the primary outcome of the study.

### Explanatory variable

Data included demographics (age & gender), reported number of AIDS cases, and incubation time.

### Data management and statistical analysis

Data were analysed using Excel 2016, EXCELSTAT 2016, and MATLAB. Fitted distributions for the total incubation time of HIV and gender includes Weibull distribution (1, 2 and 3), Gamma distribution (1, 2 and 3), log-normal, logistic, and Fisher-Tippett (1 and 2) distributions. A two-tailed p-value of <0.01 was used to consider the statistical significance.

### Back calculation method

The following back calculation formula was used:

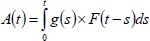

A(t)= the cumulative number of AIDS cases diagnosed by the calendar time ‘t’

g(s)= infection rate at time ‘s’

F(t-s) = the distribution function for the incubation of people who live during the period ‘s’ to ‘t’ (subjects must have been infected at some prior time).

According to the distribution fitting method, Weibull-2 distribution fitted well with the total incubation time among male and female HIV cases.

Distribution function of Weibull-2 distribution is

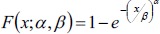


Therefore, using Weibull-2 distribution and reported number of HIV cases, we have estimated the cumulative number of AIDS cases with the back calculation method for total as well as male and female cases separately using the above mentioned equation. The first reported case of HIV in Kerala was in 1987 which was coded as t=0 for that year and then added numbers consecutively as t=1 for 1988 and so on. We have used the data of new HIV positive cases per year [g(s)] from 2005 (t=18) to 2015 (t=18).

## Results

### Fitting of parametric distributions in incubation time

The incubation time was categorised into three groups: (a) total; (b) male; and (c) female. The best fitted separate distributions were found for each group. Normality test was used to assess the nature of data, whether parametric or non-parametric (Table 1). All three groups followed a normal distribution pattern. Hence, the Chi-square test was used to establish the best fitted distribution.

The Weibull-2 distribution was the best fitted distribution for the incubation time of HIV total, male and female cases among all the distributions. Second-best fitted distribution was Gamma distribution and third-best was lognormal. The fitting of Weibull-2, Gamma family, logistic, lognormal, and Fisher-Tippett distributions are shown in Table 1. Weibull and Gamma families were the most commonly used distributions in this area.

Distribution function of Weibull (2) distribution for total HIV cases is:

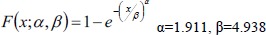


Distribution function of Weibull (2) distribution for male HIV cases is:

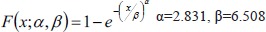


Distribution function of Weibull (2) distribution for female HIV cases is:

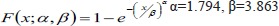


### Back calculation of cumulative AIDS cases

After fitting all appropriate distributions on incubation time of HIV patients, the Weibull-2 distribution was found to fit the best along with parameter estimation (Table 2). The mean incubation time for total (both gender), male and female HIV cases was found to be 4.4, 5.8 and 3.4 years, respectively. The year-wise cumulative number of AIDS cases among Keralites was estimated using the Weibull-2 distribution function and infection rate with the back calculation method. Table 3 shows the estimated cumulative total, male and female AIDS cases in Kerala. From the year 2005 to 2015, the estimated total cumulative AIDS cases were: 35,777, 48,944, 62,039, 45,669, 45,668, and 43,605, 42,377, 39,362, 37,617, 39,583, 25,414 respectively using back-calculation method with Weibull-2 incubation time distribution. A decreasing trend of cumulative AIDS cases has been observed during the recent years.

## Discussion

### Back calculation method

To date, research investigators have proposed several mathematical models which are often based on assumptions about the HIV prevalence and prognosis (survival rates after infection). These include dynamical models, demographic models, back-calculation techniques and birth-cohort methods [13-15]. The UNAIDS/WHO recommended EPP Impact Model which is being used in more than 120 countries worldwide for generating estimates of national HIV prevalence, incidence, mortality and treatment needs [16]. Of note, back-calculation techniques have been used mainly in low-income countries with reliable data on the number of AIDS cases diagnosed over time and also the distribution of the incubation period [17]. To the best of our knowledge the present study is unique in Kerala; it investigates the best fitted statistical distribution for incubation time of HIV and estimation of cumulative AIDS cases among Keralites using the back-calculation method.

### Estimation of cumulative number of AIDS cases

According to NACO, adult HIV prevalence has a declining trend over the years in Kerala state of India [2,18]. Consistent with these findings the present study also estimated a decrease in cumulative number of AIDS cases in Kerala from 2007 (62,039 cases) to 2015 (25,414 cases). The current study utilized different models such as the Weibull, Gamma, logistic, Fisher-Tippett, and log normal models for estimation of AIDS incubation time. Specifically, among these models, we fitted three distributions each for Weibull (1,2 and 3) and Gamma (1,2 and 3) families. Interestingly, Weibull-2 distribution was found to be the best fitted model among all the other distributions for total, male and female incubation time. This could be due to the fact that Weibull-2 distribution function is best suited to the nature of AIDS incubation time.

Weibull and Gamma distribution models are the frequently used incubation time distributions considered for the back-calculation approach. The Weibull distribution represents the proportional hazard as well as accelerated failure time model. In the late 20th century, Weibull distribution was used to study the incubation period distribution for transfusion associated AIDS cases [19,20]. Moreover, several distribution models are also proposed for the estimation of HIV incubation period. An earlier study utilized gamma, Weibull and lognormal models for estimation of HIV incubation time in patients from San Francisco [21]. Medley et al. [20] estimated a median incubation period of HIV to be 4.3 years which is in agreement with our findings of 4.4 years using the Weibull model. Whereas, Munoz and Xu reported a higher median incubation period of 7.5 years using fitted Weibull model [23]. In-line with our observation, other investigators have recommended the Weibull model as best fit for HIV incubation period [24,25]. Gamma distribution is another crucial parametric method used to model incubation period of HIV/AIDS [21,22,26]. Also, some studies utilized the log-logistic and generalized exponential model for estimating the HIV incubation period [21,22]. In addition, the log-normal distribution has been employed for HIV incubation period which fits better than the Weibull model [21]. Another approach is a staged-Markov model to assess the distribution and mean duration of incubation period among HIV infected individuals [27]. Therefore, the integration of different exponential distribution has potential to be considered for a robust HIV incubation model.

Despite, the variety of modelling approaches only a few models are fitting well with HIV incubation time. The current study suggested Weibull-2 model as most appropriate for our population. The projected AIDS estimates obtained using the Weibull incubation time density and back calculation method seems to be most plausible. In the present study, a decreasing trend of cumulative AIDS cases has been observed during the recent years. This could be explained by the fact that globally, UNAIDS has expanded its antiretroviral therapy (ART) programmes effectively. There is an increasing trend on the people receiving ART from 2000 (< 1 million) through June 2016 (18.2 million) to a projected 30 million target in 2020 [28]. In 2016, the UNAIDS made available US$ 19.1 billion towards AIDS management for nations under low-and-middle income group. Notably, UNAIDS projected to spend an estimated fund of US$ 26.2 billion for 2020 and US$ 23.9 billion for 2030 on ART programmes in these regions [27]. Evidence from this study can be used for the understanding of the disease burden, ART planning and allocation of funds for better therapeutic intervention and outcomes.

#### Limitation of the study

The present study has certain limitations. First, the back-projection model considered estimates of HIV incubation period distribution from a small-scale study. Secondly, it is to be noted that these estimates are derived from the unadjusted information on AIDS incidence. Therefore, such estimates are probably not the true representation of AIDS cases in Kerala for the studied duration. Possibly, the actual frequency of AIDS cases in Kerala might be higher than our estimates and so there is a chance of under-representation of the possible number of AIDS cases. Thus, it is unclear to what level the HIV incubation period distribution represents the epidemic scenario of HIV in Kerala. Moreover, the assessment using the parametric back-calculation is highly dependent on the incubation time parameters.

## Conclusion

It is the first study of the parametric distribution fitting of HIV incubation time among Keralites, and Weibull distribution appears to offer the best fit model. The mean incubation time of HIV is 4.4 years which showed a rapid progression of the disease in people living with HIV in Kerala. This is most likely attributed to the late presentation and diagnosis of AIDS-related conditions in the early phase of an epidemic and a delayed therapeutic intervention. This study reveals that back-calculation method is a powerful tool to estimate the cumulative AIDS cases of Kerala which shows a decreasing trend.

### Future scope of the study

It is advisable to check the applicability of the stratified models to characterise the behaviour and onset of the HIV epidemic based on age and gender. These models will imitate the infection curve for the respective districts or geographical regions, model using data at lower hierarchical levels, spatiotemporal model that predict HIV prevalence rates based on the time factor in locations of the state, Bayesian models, and sexual networks models.

### What is already known on this topic

Mean incubation time of HIV in selected states of India.

### What this study adds

Mean incubation time of HIV in Kerala. Applicability of Weibull-2 distribution for HIV data in Kerala.

## Figures and Tables

**Figure 1:**
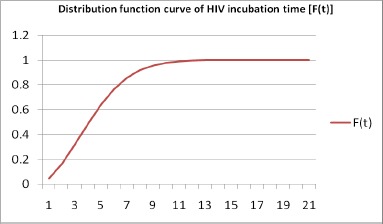
Weibull-2 distribution function probability of HIV incubation time (years)

**Table 1:** Fitting of distributions for incubation time of total, male & female HIV cases

Distribution	P value		
	Total cases	Male	Female
**Weibull (1)**	< 0.01	< 0.01	< 0.01
**Weibull (2)**	0.054*	0.035*	0.018*
**Weibull (3)**	< 0.01	0.022*	0.033*
**Gamma (1)**	< 0.01	0.059*	< 0.01
**Gamma (2)**	< 0.01	0.036*	< 0.01
**Gamma (3)**	< 0.01	< 0.01	< 0.01
**Log-normal**	0.011*	0.032*	0.0001
**Logistic**	0.032*	0.017*	0.022*
**Fisher- Tippet (1)**	< 0.01	< 0.01	< 0.01
**Fisher- Tippet (2)**	0.010*	0.030*	< 0.01

* p> 0.01- Sample follows the distribution. H0: Sample follows the distribution, HA: Sample does not follow the distribution

**Table-2:** Distribution fitting of Weibull-2 distribution for incubation time of total, male and female HIV cases

Variables	Total cases		Male		Female	
**Parameter**	**Value**	**Standard error**	**Value**	**Standard error**	**Value**	**Standard error**
**Alpha**	1.911	0.329	2.831	0.718	1.794	0.428
**Beta**	4.938	0.576	6.508	0.813	3.863	0.623
**Statistic**	**Data**	**Parameters**	**Data**	**Parameters**	**Data**	**Parameters**
**Mean**	4.409	4.381	5.778	5.797	3.462	3.436
**Variance**	5.652	5.693	5.444	4.926	3.884	3.926
**Skewness (Pearson)**	0.366	0.693	0.552	0.226	-0.133	0.784
**Kurtosis (Pearson)**	-0.169	0.367	-1.324	-0.244	-1.145	0.569

**Table 3:** Year-wise total cumulative AIDS cases in Kerala

Year	Total	Male	Female
**2005**	35,777	18,011	16,763
**2006**	48,944	24,530	23,191
**2007**	62,039	31,913	28,573
**2008**	45,669	25,115	19,250
**2009**	45,668	24,952	19,529
**2010**	43,605	24,118	18,390
**2011**	42,377	23,918	17,397
**2012**	39,362	21,795	16,691
**2013**	37,617	22,950	13,629
**2014**	39,583	22,602	16,118
**2015**	25,414	15,298	9,506
